# Indications and Findings of Upper Endoscopies in Males and Females, Are They the Same or Different?

**DOI:** 10.3390/jcm10081620

**Published:** 2021-04-11

**Authors:** Naim Abu-Freha, Roni Gat, Aerin Philip, Baha Yousef, Liza Ben Shoshan, David Yardeni, Anat Nevo-Shor, Victor Novack, Ohad Etzion

**Affiliations:** 1 Soroka University Medical Center, Faculty of Health Sciences, The Institute of Gastroenterology and Hepatology, Ben-Gurion University of the Negev, Beer-Sheva 84101, Israel; bahayo@clalit.org.il (B.Y.); lizana1@clalit.org.il (L.B.S.); yardeda@gmail.com (D.Y.); anatnev@gmail.com (A.N.-S.); ohadet34@yahoo.com (O.E.); 2 Soroka Clinical Research Center, Soroka University Medical Center, Faculty of Health Sciences, Ben-Gurion University of the Negev, Beer Sheva 84010, Israel; roniigat@gmail.com (R.G.); victorno@clalit.org.il (V.N.); 3 Faculty of Health Sciences, Medical School for International Health, Ben-Gurion University of the Negev, Beer-Sheva 84101, Israel; aerin@post.bgu.ac.il

**Keywords:** upper endoscopy, males, females, indication, findings

## Abstract

Sex and gender can affect the prevalence and prognosis of diseases. Our aim was to assess similarities and differences for males and females who underwent an upper endoscopy, with regards to indications and results. We reviewed all upper endoscopy reports from 2012 to 2016. Data regarding demographics, indications, and procedure findings were collected. The upper endoscopy findings were compared regarding the most common indications: gastroesophageal reflux, abdominal pain, gastrointestinal bleeding, and anemia. We investigated 12,213 gastroscopies among males (age, 56.7 ± 17.4) and 15,817 among females (age, 56.0 ± 17.3, *p* = 0.002). Males who underwent an upper endoscopy for gastroesophageal reflux had higher rates of esophagitis (7.7% vs. 3.4%, *p* < 0.001) and Barret’s esophagus (4.4% vs. 1.5%, *p* < 0.001). Females who underwent an upper endoscopy for abdominal pain had a higher rate of hiatal hernia, whereas males had higher rates of esophagitis, helicobacter pylori infection, gastritis, gastric ulcer, duodenitis, and duodenal ulcer (*p* < 0.001). Gastrointestinal bleeding as an indication for upper endoscopy showed that helicobacter, duodenitis, and duodenal ulcers are more common among males compared to females (*p* < 0.001). Males with anemia who underwent an upper endoscopy had higher rates of esophagitis (*p* = 0.021) gastritis (*p* = 0.002), duodenitis (*p* < 0.001), and duodenal ulcer (*p* < 0.001). We found significant differences regarding the pathological gastroscopy findings between males and females in relation to the different indications.

## 1. Introduction

Within the last two decades, there has been an emergence of research interest concerning the impact that sex and gender have on diseases. Sex defines the biological or anatomical variance between males and females, whereas gender differentiates the social roles and cultural norms of men and women.

Prevalence of the disease, symptoms, severity, and outcome differences in some diseases were previously reported. Sex differences can be attributed to genetics, hormones, body structure, and physiological factors. Throughout the past decades, gender has evolved; both genders have changed in their social roles and types of employment. There is a recognizable increase in work hours among women, contributing additional psychosocial stress in the midst of established childcare responsibilities [1,2].

One of the investigated fields is cardiovascular disease, which shows there is a higher incidence of heart attacks or fatal coronary heart disease among men compared to women [3]. In the gastroenterology field, colorectal cancer (CRC) is a common cancer among both genders and has been investigated according to the differences between females and males. Sex-associated differences were found in CRC development including incidence, anatomical site, and survival [4,5]. Women have higher rates of right colon cancer, and right and left colon cancers differ in their developmental pathways. Hormonal, genetic, and environmental factors were identified as contributors to the differences in colorectal cancer between males and females [4,5].

Endoscopy is an important diagnostic and therapeutic modality in gastroenterology. Until now, there has been scarce published data regarding the differences in indications and findings between males and females. The aim of the present study is to assess the similarities and differences in those who have undergone esophagogastroduodenoscopy (EGD) according to their sex and each specified common indication for endoscopy.

## 2. Materials and Methods

### 2.1. Patients

In this retrospective study, we included data of all consecutive patients who underwent EGD between the years of 2012–2016 in the Department of Gastroenterology and Liver Disease at Soroka University Medical Center (SUMC). SUMC is a tertiary, 1100-bed hospital located in the city of Beer-Sheva in southern Israel. It is the only medical center providing tertiary care to a population of approximately 700,000 residents. The Department of Gastroenterology and Liver Disease provides endoscopy services for hospitalized patients as well as ambulatory gastrointestinal endoscopic services and medical care for the largest Health Maintenance Organizations (HMOs) in southern Israel.

### 2.2. Data Collection

Demographic and clinical data for all patients were gathered and reviewed using a computerized database. Demographic data collected from patient medical records included age, sex, indications, and results of the first EGD; control examinations were excluded. The most common four indications were chosen: gastroesophageal reflux (GERD), abdominal pain, gastrointestinal bleeding and anemia. Helicobacter pylori testing, via urease fast test, was collected as reported in the EGD reports. Diagnosis of gastritis, duodenitis, and peptic ulcer were performed by endoscopy, whereas diagnosis of malignancy was made by both endoscopy and histology. Endoscopy data was analyzed and compared between males and females. The study was carried out in accordance with the principles of the Helsinki Declaration. The study protocol was approved by the Institutional Ethical Committee.

### 2.3. Statistical Analysis

Patient characteristics were presented as mean ± SD for continuous variables and as percentages for categorical variables. Categorical variables were compared using the chi-square test or Fisher’s exact test. Continuous variables were examined with the student t-test. Continuous variables that were not normally distributed were reported as median (IQR) and compared in the Kruskal–Wallis test. All statistical analyses were performed using IBM SPSS version 24 (Chicago, IL, USA). *p*-values less than 0.05 were considered statistically significant.

## 3. Results

Our study included 28,030 EGD, with 12,213 (43,6%) procedures performed in males. The mean age of males was 56.7 ± 17.4 years vs. 56.0 ± 17.3 years in females (*p* = 0.002). The most common indications for EGD were gastroesophageal reflux, abdominal pain, gastrointestinal bleeding, and anemia. The findings of EGD among all patients included in this cohort are presented in Table 1. The findings of EGD performed due to these indications were compared between males and females and are presented in Table 2, Table 3, Table 4 and Table 5.

Females who underwent EGD for GERD symptoms had significantly more rates of hiatal hernia (41.7% vs. 26.8%, *p* < 0.001), while males had more duodenitis and helicobacter infections (9.5% vs. 4.7%, *p* < 0.001 and 4.1% vs. 1.9%, *p* = 0.011; respectively).

7845 (59.5%) of the included females had abdominal pain as an indication for EGD compared with 4342 (35%) males. Males who underwent EGD for abdominal pain had more esophageal ulcer, esophageal polyp, gastritis, helicobacter pylori infection, duodenitis, and duodenal ulcer (*p* < 0.001) than females who underwent EGD for the same indication. In contrast, women had more hiatal hernia (25.9% vs. 10.3%, *p* < 0.001) and a higher proportion of normal EGD (42.8% vs. 38.7%, *p* < 0.001).

Only 632 (4%) females underwent EGD for gastrointestinal bleeding compared with 1170 (9.6%) males. Duodenitis and duodenal ulcer were significantly more common among males who underwent EGD for anemia or gastrointestinal bleeding compared to females (*p* < 0.001). Gastritis was more common among males who underwent EGD for anemia but not for gastrointestinal bleeding.

In Table 6, the relative risks for abnormal findings among females in gastroscopy according to the different indications are summarized. Hiatal hernia was more common among females in all four indications, and there was a higher proportion for normal EGD among females with abdominal pain or gastrointestinal bleeding.

## 4. Discussion

The present study is, to the best of our knowledge, the first one that investigates differential upper endoscopy indications and findings among women and men. The major finding of our study is that significant sex differences exist in EGD findings related to their indications for EGD.

Our study is the first one to investigate specific symptoms, and we found that hiatal hernia is more common among females independent of the EGD indications. However, there was more prominence of reflux symptoms among females who underwent EGD. The high rate of hiatal hernia among females in our study was related to the specific indications. This higher rate can be explained by several factors including high intra-abdominal pressure from previous pregnancy, obesity, or hormonal factors. However, to elucidate the exact causes and risk factors for the higher rate of hiatal hernia among females requires further prospective studies. Compared with females, gastritis was more common among males with abdominal pain and anemia, but there is no significant difference among males with GERD symptoms or gastrointestinal bleeding. Gastric ulcer had higher prevalence among males with abdominal pain and there was no difference in other indications including GERD, anemia, or gastrointestinal bleeding. Duodenitis was significantly higher among males with different indications. No difference regarding duodenal ulcer was found in the indication of GERD among males, but there was a higher frequency of duodenal ulcer found in the other three indications including abdominal pain, anemia, and gastrointestinal bleeding. In addition, helicobacter infection was more common among males with GERD, abdominal pain, and gastrointestinal bleeding but no significant difference was noted between males and females with anemia. No significant difference was found regarding gastric carcinoma in males and females related to the specific indications.

There has been an increasing interest observed within the past few decades regarding differences in disease presentations and outcomes due to biological differences between males and females. Generally, differences can be explained by the variation in body structure; hormone disparity; and prevalence of unique, sex-based risk factors. Additionally, comorbidity of diseases such as diabetes mellitus, obesity, and autoimmune diseases have systemic effects on the gastrointestinal tract, and these diseases have differing prevalence between females and males.

The body structure of females and males are different. Differences in fat storage, fat metabolism, and health risks of obesity among females and males were noted [6]. Generally, female bodies are pear-shaped structures and male bodies are apple-shaped structures. Abdominal obesity and wider waists can contribute directly to reflux symptoms, thereby increasing the prevalence of GERD, which further increases the risk for Barrett’s esophagus and esophageal carcinoma. Male predominance of esophageal cancer is strongly associated with body fat distribution, mentioned above as apple-shaped [7].

Some risk factors are more common among men, these risk factors can contribute to the different frequency of specific indications and pathologies found in upper endoscopies. Smoking rates are generally higher in men and can influence the development of gastrointestinal pathologies. Sex-dependent obesity prevalence varies by geographical region: in some countries, obesity is higher among men compared to women; in other countries, an opposite trend is observed [8,9,10,11,12]. Multiple studies have shown that obesity, especially abdominal and visceral obesity, is a risk factor for GERD, Barrett’s esophagus, and esophageal adenocarcinoma [13]. Furthermore, gender differences exist with regard to eating habits and preferences; women generally consume healthier foods including foods high in added sugars and dairy, whereas men generally prefer energy-dense processed food such as ice cream, chocolate, and cookies [14,15,16]. Alcohol consumption is higher among men [17]. All the abovementioned risk factors including smoking, obesity, and alcohol consumption can influence the frequency of the different indications and pathological findings of upper endoscopies among males and females.

Another important factor is hormones. Estrogen is an important sex hormone that not only regulates female reproductive functions but also contributes to several biological functions including protection from different diseases. Focusing on the gastrointestinal tract, estrogen’s role in the pathophysiology of different diseases has been found, including GERD, esophageal cancer, peptic ulcers, gastric cancer, inflammatory bowel disease, irritable bowel syndrome, and colon cancer [18,19,20,21]. Estrogen was described to have a protective effect on the development of esophageal cancer, peptic ulcer, and gastric carcinoma [18]. Contrary to our results, a lower rate of esophagitis (GERD) was found among females. However, there was an insignificant difference between males and females regarding peptic ulcers or gastric cancer. Additional studies are needed to focus on the effect of estrogen on endoscopy findings.

Female predominance was clearly observed in different autoimmune diseases [22]. In our study, significant higher prevalence of celiac disease as indication for upper endoscopy was found in females compared to males (data not included), which correlates with the higher prevalence for celiac disease in females [22].

In addition, it is important to mention that age may affect the prevalence of endoscopic lesions, a significant age difference between females and males was found in our cohort related to the indications GERD and upper gastrointestinal bleeding and anemia. As previously reported, there is an increase in the prevalence of GERD and esophagitis with aging, particularly among females [19]. Additional studies investigating the prevalence of lesions found in EGD in different aging periods are needed.

To summarize, the present study showed significant differences regarding EGD findings among males and females related to the most common indications. Hiatal hernia was significantly more prevalent among females in all common included EGD indications. In addition, peptic ulcers, inflammation, and helicobacter pylori infection were more common among males then females. Inflammation of the stomach or duodenum with the development of peptic ulcers may derive from risk factors such as higher rates of smoking and helicobacter infections among males [23]. No significant difference was found regarding gastric carcinoma related to the specific indication. The findings of the study may provide gastroenterologists with explanations of sex differences in their physician–patient interactions, particularly regarding the specific indication investigated in the present study. Additionally, after confirmation of our findings, other variables such as age and comorbidities can be added and combined to this understanding with the potential to develop a prediction model that estimates the risk of abnormal gastroscopy findings. This tool would benefit gastroenterologists in making decisions for endoscopy referrals. The strengths of this study include the fact that its findings derive from a large number of EGDs, reaching nearly 28,000 procedures, and that it is the first study that has investigated the upper endoscopy procedure with regard to sex differences. The study also has several limitations including its retrospective design, lack of long-term outcomes, lack of data regarding treatment with proton pump inhibitors, and the fact that biopsies and/or urease quick test were not performed in every EGD.

## 5. Conclusions

Significant differences in the pathological findings of upper endoscopies were found between males and females in this study. Future research in this field should focus on the identification of factors contributing to these differences and the development of sex-specific approaches for diagnosis and management of gastrointestinal diseases.

## Figures and Tables

**Table 1:** Upper endoscopy findings among all included cohorts.

	Male *n* = 12,213 (%)	Female *n* = 15,817 (%)	*p*-Value
Normal EGD	4744 (38.8)	6741 (42.6)	<0.001
Hiatal Hernia	1733 (14.0)	4999 (31.2)	<0.001
Achalasia	85 (0.7)	58 (0.4)	<0.001
Esophagitis	1032 (8.5)	715 (4.5)	<0.001
Barrett’s Esophagus	379 (3.1)	192 (1.2)	<0.001
Malignancy of Esophagus	17 (0.14)	12 (0.07)	0.55
Gastritis	4107 (33.2)	4762 (29.7)	<0.001
Gastric Ulcer	694 (5.6)	554 (3.5)	<0.001
Gastric Carcinoma	45 (0.4)	29 (0.2)	0.003
Polyp of Stomach	755 (1.6)	1219 (7.6)	<0.001
Duodenitis	1195 (16.1)	1177 (7.4)	<0.001
Duodenal Ulcer	754 (6.1)	367 (2.3)	<0.001

EGD: Esophagogastroduodenoscopy.

**Table 2:** Upper endoscopy findings for the indication of gastroesophageal reflux.

	Gastroesophageal Reflux		
Findings	Male (*n* = 639)	Female (*n* = 937)	*p*-Value
Age, Mean ± SD	53.2 ± 16.1	57.7 ± 14.2	<0.001
Hiatal Hernia	171 (26.8)	391 (41.7)	<0.001
Esophagitis	49 (7.7)	32 (3.4)	<0.001
Esophageal Ulcer	2 (0.3)	3 (0.3)	0.980
Barret’s Esophagus	28 (4.4)	14 (1.5)	<0.001
Gastritis	117 (18.3)	189 (20.2)	0.359
Gastric Ulcer	5 (0.8)	11 (1.2)	0.447
Gastric Carcinoma	1 (0.2)	0 (0.0)	N/A
Duodenitis	61 (9.5)	44 (4.7)	<0.001
Duodenal Ulcer	5 (0.8)	6 (0.6)	0.739
Helicobacter pylori infection	26 (4.1)	18 (1.9)	0.011
Normal	208 (32.6)	315 (33.6)	0.659

**Table 3:** Upper endoscopy findings for the indication of abdominal pain.

Indication/Finding	Abdominal Pain		
	Male (*n* = 4342)	Female (*n* = 7845)	*p*-Value
Age, Mean ± SD	52.1 ± 17.1	52.5 ± 17.0	0.081
Hiatal Hernia	447 (10.3)	2033 (25.9)	<0.001
Esophagitis	223 (5.1)	163 (2.1)	<0.001
Barret’s Esophagus	48 (1.1)	33 (0.4)	<0.001
Esophageal Ulcer	18 (0.4)	8 (0.1)	<0.001
Esophageal Polyp	23 (0.5)	15 (0.2)	<0.001
Gastritis	1345 (31.0)	2042 (26.0)	<0.001
Gastric Ulcer	59 (1.4)	70 (0.9)	0.016
Gastric Carcinoma	5 (0.1)	7 (0.1)	0.662
Duodenitis	716 (16.5)	478 (6.1)	<0.001
Duodenal Ulcer	125 (2.9)	83 (1.1)	0.001
Helicobacter pylori infection	320 (7.4)	363 (4.6)	<0.001
Gastric MATL-lymphoma	9 (0.2)	4 (0.05)	0.011
Normal	1679 (38.7)	3356 (42.8)	<0.001

MATL: Mucosa associated lymphoid tissue.

**Table 4:** Upper endoscopy findings for the indications of anemia.

	Anemia		
	Male (*n* = 1466)	Female (*n* = 1575)	*p*-Value
Age, Mean ± SD	65.3 ± 14.1	63.6 ± 15.6	0.016
Hiatal Hernia	161 (11.0)	411 (26.1)	<0.001
Esophagitis	52 (3.5)	34 (2.2)	0.021
Barret’s Esophagus	23 (1.6)	8 (0.5)	0.004
Esophageal Ulcer	9 (0.6)	3 (0.2)	0.063
Gastritis	342 (23.3)	296 (18.8)	0.002
Gastric Ulcer	36 (2.5)	38 (2.4)	0.939
Gastric Carcinoma	7 (0.5)	3 (0.2)	0.167
Duodenitis	136 (9.3)	60 (3.8)	<0.001
Duodenal Ulcer	57 (3.9)	26 (1.7)	<0.001
Helicobacter pylori infection	52 (3.5)	41 (2.6)	0.131
Normal	562 (38.3)	620 (39.4)	0.561

**Table 5:** Upper endoscopy finding for the indication of gastrointestinal bleeding.

Indication/Finding	Upper Gastrointestinal Bleeding		
	Male (*n* = 1170)	Female (*n* = 632)	*p*-Value
Age, Mean ± SD	63.6 ± 17.2	67.5 ± 16.4	<0.001
Hiatal Hernia	69 (5.9)	80 (12.7)	<0.001
Esophagitis	70 (6.0)	34 (5.4)	0.600
Barret’s Esophagus	3 (0.3)	2 (0.3)	0.817
Esophageal ulcer	29 (2.5)	12 (1.9)	0.431
Gastritis	216 (18.5)	105 (16.6)	0.328
Gastric Ulcer	150 (12.8)	70 (11.1)	0.280
Gastric Carcinoma	10 (0.9)	3 (0.5)	0.363
Duodenitis	155 (13.2)	49 (7.8)	<0.001
Duodenal Ulcer	220 (18.8)	82 (13.0)	0.002
Helicobacter Pylori infection	60 (5.1)	15 (2.4)	0.005
Gastric MATL-lymphoma	2 (0.17)	1 (0.15)	0.894
Normal	157 (13.4)	127(20.1)	<0.001

**Table 6:** The relative risk (for female) for findings in gastroscopy.

	Indication	Anemia			Gastro Bleed			Reflux			Abdominal Pain		
Diagnosis		RR	95% CI	*p*	RR	95% CI	*p*	RR	95% CI	*p*	RR	95% CI	*p*
Hiatal Hernia		2.86	2.35, 3.50	<0.001	2.31	1.61, 3.24	<0.001	1.96	1.58, 2.44	<0.001	3.05	2.73, 3.40	<0.001
Esophageal Polyp		1.86	0.47, 7.47	0.379	1.23	0.21, 7.41	0.817	N/A			0.36	0.19, 0.69	0.002
Gastritis		0.76	0.64, 0.91	0.002	0.88	0.68, 1.14	0.328	1.13	0.87, 1.46	0.359	0.78	0.72, 0.85	<0.001
Helicobacter Pylori		0.73	0.48, 1.10	0.132	0.45	0.25, 0.80	0.006	0.46	0.25, 0.85	0.013	0.61	0.52, 0.71	<0.001
Gastric Ulcer		0.98	0.62, 1.56	0.939	0.85	0.63, 1.15	0.281	1.51	0.52, 4.36	0.450	0.65	0.46, 0.93	0.017
Gastric Carcinoma		0.40	0.09, 1.43	0.182	0.55	0.15, 2.02	0.370	N/A			0.77	0.25, 2.44	0.663
Duodenitis		0.39	0.28, 0.53	<0.001	0.55	0.39, 0.77	0.001	0.47	0.31, 0.70	<0.001	0.33	0.29, 0.37	<0.001
Duodenal Ulcer		0.41	0.26, 0.66	<0.001	0.64	0.49, 0.85	0.002	0.82	0.25, 2.69	0.740	0.36	0.27, 0.48	<0.001
Normal		1.04	0.90, 1.21	0.561	1.62	1.25, 2.10	<0.001	1.05	0.85, 1.30	0.659	1.19	1.10, 1.13	<0.001

RR: relative Risk; CI: Confidence interval.

## Data Availability

No additional data are available.
